# Initial Despair and Current Hope of Identifying a Clinically Useful Treatment of Myocardial Reperfusion Injury: Insights Derived from Studies of Platelet P2Y_12_ Antagonists and Interference with Inflammation and NLRP3 Assembly

**DOI:** 10.3390/ijms25105477

**Published:** 2024-05-17

**Authors:** Michael V. Cohen, James M. Downey

**Affiliations:** 1 The Departments of Physiology and Cell Biology, Frederick P. Whiddon College of Medicine, Mobile, AL 36688, USA; jdowney@southalabama.edu; 2 The Departments of Medicine, Frederick P. Whiddon College of Medicine, Mobile, AL 36688, USA

**Keywords:** cangrelor, caspase-1, myocardial infarction, NLRP3 inflammasome, P2Y_12_ antagonists, reperfusion injury, VX-765

## Abstract

Myocardial necrosis following the successful reperfusion of a coronary artery occluded by thrombus in a patient presenting with ST-elevation myocardial infarction (STEMI) continues to be a serious problem, despite the multiple attempts to attenuate the necrosis with agents that have shown promise in pre-clinical investigations. Possible reasons include confounding clinical risk factors, the delayed application of protective agents, poorly designed pre-clinical investigations, the possible effects of routinely administered agents that might unknowingly already have protected the myocardium or that might have blocked protection, and the biological differences of the myocardium in humans and experimental animals. A better understanding of the pathobiology of myocardial infarction is needed to stem this reperfusion injury. P2Y_12_ receptor antagonists minimize platelet aggregation and are currently part of the standard treatment to prevent thrombus formation and propagation in STEMI protocols. Serendipitously, these P2Y_12_ antagonists also dramatically attenuate reperfusion injury in experimental animals and are presumed to provide a similar protection in STEMI patients. However, additional protective agents are needed to further diminish reperfusion injury. It is possible to achieve additive protection if the added intervention protects by a mechanism different from that of P2Y_12_ antagonists. Inflammation is now recognized to be a critical factor in the complex intracellular response to ischemia and reperfusion that leads to tissue necrosis. Interference with cardiomyocyte inflammasome assembly and activation has shown great promise in attenuating reperfusion injury in pre-clinical animal models. And the blockade of the executioner protease caspase-1, indeed, supplements the protection already seen after the administration of P2Y_12_ antagonists. Importantly, protective interventions must be applied in the first minutes of reperfusion, if protection is to be achieved. The promise of such a combination of protective strategies provides hope that the successful attenuation of reperfusion injury is attainable.

## 1. Acute Myocardial Infarction Involves Thrombus Formation

Myocardial infarction (MI) has been recognized for centuries as an often fatal disorder. We now recognize that, even for those individuals not succumbing to the disease, many will progress to congestive heart failure because of the loss of sufficient contractile myocardium from the ensuing necrosis. Despite these apparent and frequent clinical events, the poor understanding of the pathophysiology of MI has limited scientific inquiry and specific intervention. Multiple early investigations of the nature of transmural MI presenting with ECG evidence of S-T segment elevation depended on observational autopsy studies, which were often handicapped by the variability of intervals between the clinical event and pathologic examination [1]. Whereas some noted that a high percentage of patients that died after infarction had total coronary artery occlusion, many others observed that only 50% or fewer had a total occlusion and concluded that thrombosis was a secondary event rather than the primary causation. But, in a landmark study in 1980, DeWood and colleagues [2] evaluated the coronary angiograms of 322 patients hospitalized within twenty-four hours of a transmural MI. Notably, 110 of 126 patients (87%) in whom an angiogram was performed within four hours of symptom onset were noted to have a total coronary occlusion of the infarct artery. When the interval between the onset of symptoms and angiography ranged, instead, from twelve to twenty-four hours (57 patients), only 65% displayed a total occlusion of the coronary artery (*p* < 0.01, vs. 0–4 h group). Hence, it became dogma that transmural or S-T segment elevation MI (STEMI) was caused by the complete occlusion of a coronary artery. It also became apparent that the failure of earlier autopsy studies to identify this vascular occlusion as the proximate and consistent cause of infarction was related to the variable interval between symptom onset and pathologic examination, which encouraged antemortem recanalization and/or postmortem thrombolysis and thrombus retraction from the arterial wall.

Now armed with the knowledge that STEMI was caused by coronary artery thrombosis, an effective intervention could be designed to interfere with thrombosis and subsequent infarction. The early observations suggested that, at the site of the coronary thrombus, there was an ulcerated atheroma where the overlying fibrous cap had ruptured following the macrophage release of interstitial collagenases and elastolytic proteases, thus exposing blood elements to the lipid-rich, necrotic atherogenic core [3,4]. Platelets could then aggregate, and circulating coagulation proteins gained access to tissue factor-associated macrophages, resulting in the formation of a red thrombus. More recently, the increasing contribution of superficial coronary erosion to the generation of acute coronary syndrome has been recognized [3]. The erosion characterized by apoptotic endothelial cell death and the separation of endothelial cells from the underlying basement membrane following nonfibrillar collagen degradation were associated with atheroma-caused increased shear stress. Platelets could adhere to the exposed subendothelium, aggregate, and release their granular contents, leading to the formation of a platelet-rich white thrombus. Anti-platelet aggregatory drugs are likely to be much more effective in this latter type of thrombus. But the distinction between thrombi caused by plaque rupture and superficial erosion was not appreciated in the 1980s. A ubiquitous agent known to impede platelet aggregation was acetylsalicylic acid (ASA) or aspirin, which irreversibly blocks cyclooxygenase-1 within platelets to inhibit the production of the aggregatory thromboxane A_2_ [5]. Multiple investigations of the efficacy of ASA were initiated [6,7,8]. The initial results were encouraging. Large-scale meta-analyses of numerous published studies demonstrated the ability of ASA to significantly decrease vascular complications and cardiac deaths by 15–30% in both acute and chronic coronary syndromes [7,8]. In fact, after the establishment of ASA’s efficacy, all MI patients have been treated with ASA. Consequently, ASA became the standard treatment for STEMI, and all subsequent clinical evaluations of newer pharmacologic and mechanical interventions for the treatment of ischemic heart disease have been conducted in the presence of ASA. As discussed below, this practice may have had profound consequences.

## 2. The Advent of P2Y_12_ Antagonists

Many years after the use of ASA became the standard treatment for ischemic heart disease, a new class of anti-platelet aggregatory agents was introduced. The new drugs block adenosine phosphate (ADP) P2Y_12_ receptors on platelets [9]. There are four varieties of a platelet P2Y_12_ antagonist that have been used clinically [10]. Clopidogrel, prasugrel, and ticagrelor are oral medications, whereas cangrelor is a short-lived, intravenous preparation. Clopidogrel was the first to gain clinical acceptance as a supplement to ASA in the treatment of ischemic heart disease and as a necessary agent to prevent in-stent thrombosis after percutaneous coronary intervention (PCI). Clopidogrel is a thienopyridine, an irreversible inhibitor of platelet aggregation, and a pro-drug that requires in vivo metabolic activation in two steps to yield the active metabolite. Unfortunately, its activation by the CYP450 system may fail in patients with certain genetic polymorphisms. Furthermore, the onset of activity of oral drugs may be slow. Gastrointestinal motility may be decreased in ill patients with acute MI (AMI), and morphine, which further decreases gut motility, may complicate drug absorption [11,12]. Thus, clopidogrel may not be ideal for the acute treatment of an MI patient if one is depending on its anti-platelet effects at the time of coronary artery recanalization and stenting.

Prasugrel is activated after only one metabolic conversion, and there are no known genetic polymorphisms that affect this conversion [10]. But even a one-step metabolic conversion takes time and delays the onset of the anti-platelet effect. And the oral absorption of the drug may be delayed, as noted above.

Ticagrelor is a cyclopentyltriazolopyrimidine and is not a pro-drug [10]. Thus, it does not require metabolic conversion. Furthermore, unlike clopidogrel and prasugrel, ticagrelor is a reversible inhibitor of the P2Y_12_ platelet receptor. Because there is no need for metabolic activation, ticagrelor’s effect on platelets is more rapid than that of clopidogrel or prasugrel. However, the delayed G-I absorption of the drug may still affect the timing of its anti-platelet effect.

Finally, cangrelor is also a cyclopentyltriazolopyrimidine and needs no metabolic activation [10]. Unlike the other three P2Y_12_ antagonists, cangrelor is administered intravenously, and, therefore, the precise time of exposure of the coronary vasculature is known. The onset of action is within a minute or two.

In large clinical trials comparing the effectiveness of the combination of ASA and each of the four platelet P2Y_12_ receptor antagonists, the combination significantly decreased the composite of vascular death, MI, and stroke in 15–30% of study patients [10]. Furthermore, the speed and degree of platelet inhibition were significantly increased by ticagrelor and cangrelor compared to clopidogrel. And cangrelor has a short half-life of less than five minutes, making it unsuitable for long-term treatment but ideal for the precise timing of its anti-platelet effect and rapid washout if needed.

Anti-platelet agents spare the myocardium by interfering with thrombus formation and propagation, thus reducing the duration of ischemia. The time course of myocardial cell death is not precisely known for humans, but animal studies indicate a few cells start to die within about thirty minutes after total ischemia, and then, a core of irreversible injury spreads from those cells until the entire field supplied by the occluded artery succumbs sometime between six and twenty-four hours later [13,14].

Thus, the clinical treatment of acute and chronic ischemic heart disease with anti-platelet agents has been established as an acceptable technique to diminish the complications of coronary thrombosis. But the decreased incidence of complications in 15–30% of patients implies that 70–85% of patients are still dying or having vascular complications. There is obviously much room for improvement.

## 3. Reperfusion Injury—Is It Real and Can It Be Blocked?

The use of anti-platelet agents to interfere with thrombus formation and the total occlusion of a coronary artery was soon followed by aggressive invasive recanalization procedures, which further improved outcomes in STEMI patients. But the reperfusion of an ischemic myocardium could not be performed without evident significant infarction and loss of function. However, basic scientific investigators provided insights into the pathobiology of MI, especially during the critical period after the onset of reperfusion.

In the 1970s, Maroko [15], working in Braunwald’s research lab, developed a technique by which the effect of transient myocardial ischemia could be quantitated, enabling a precise evaluation of interventions proposed to limit myocardial ischemia and hopefully subsequent sequelae. His investigations measured the amount of S-T segment elevation evident on ECG recordings from the cardiac surface following ten-minute coronary occlusions in dogs. Then, the occlusion was removed, permitting reperfusion. A second transient coronary occlusion could be performed after the application of an experimental intervention. Maroko, Braunwald, and colleagues assumed that the sum of S-T segment shifts recorded at specified loci on the heart’s anterior surface was an easily quantified measurement of myocardial ischemia. The attenuation of this sum after an intervention was considered to be an indication that the intervention had protected the heart. Thus, importantly, each animal served as its own control. Braunwald’s lab considered that the critical process determining whether the myocardium could be salvaged or became necrotic was a finely tuned supply–demand relationship. Hence, β-blockers, which could reduce the demand, and interventions such as hyaluronidase [16], which were thought to promote oxygen delivery, were considered to be central to the goal of attenuating MI. These scientific insights fueled much laboratory investigation, which ushered in an era of expectant cardioprotective intervention.

In 1973, Hearse et al. [17] made seminal observations that introduced the concept of reperfusion injury. Whereas the prolonged perfusion of rat hearts with hypoxic buffer had little consequence, a switch back to oxygenated perfusate produced immediate cell death. Hence, the reintroduction of oxygen, which was obviously necessary for the eventual recovery of the tissue, itself caused injury. This phenomenon was labeled the “oxygen paradox”. The discovery of the oxygen paradox provided clinicians hope that ischemic myocardium could be salvaged. It was widely appreciated that, in patients presenting to hospitals after acute coronary artery thrombosis, the heart could not be reperfused soon enough to prevent the irreversible ischemic injury related to absent coronary flow. However, if much of the myocardial injury occurred at reperfusion when oxygenated blood was reintroduced into the ischemic vascular territory, it would not be too late to intervene at that time to attenuate reperfusion injury.

So, how could reperfusion injury be attenuated? It was hypothesized that the reintroduction of oxygen might produce a burst of free radicals. The latter would then in turn damage the sarcolemma, interfere with ion pumps, and produce volume dysregulation. Additionally, leukocytes would invade the reperfused myocardium and release free radicals. Lucchesi and his lab championed this concept of the importance of free radicals in MI [18]. Free radical scavengers decreased infarction in a canine model of ischemia/reperfusion. Despite the many reports from Lucchesi’s lab supporting the conclusion that free radical scavengers were cardioprotective by reducing reperfusion injury, the results from multiple other labs were inconsistent [19], resulting in sufficient doubt that free radical scavengers could cause a significant reduction in MI. The same held for the investigation of anti-inflammatory agents [20]. Most infarct studies were initially performed in dogs. Unfortunately, the infarct size was noted to be highly variable from dog to dog, despite the consistent duration of the ischemic time. This was noted to be the consequence of coronary collateralization in dogs [14]. Until this was recognized, many false positive and negative studies were published. Even if these investigations from multiple research labs had produced consistent data, the small amount of salvage of 10% would probably not have been expected to produce meaningful salutary clinical results. Many suggested that it was impossible to modify the wave of cell death during ischemia without modifying residual blood flow.

## 4. Ischemic Preconditioning

**Figure 1:**
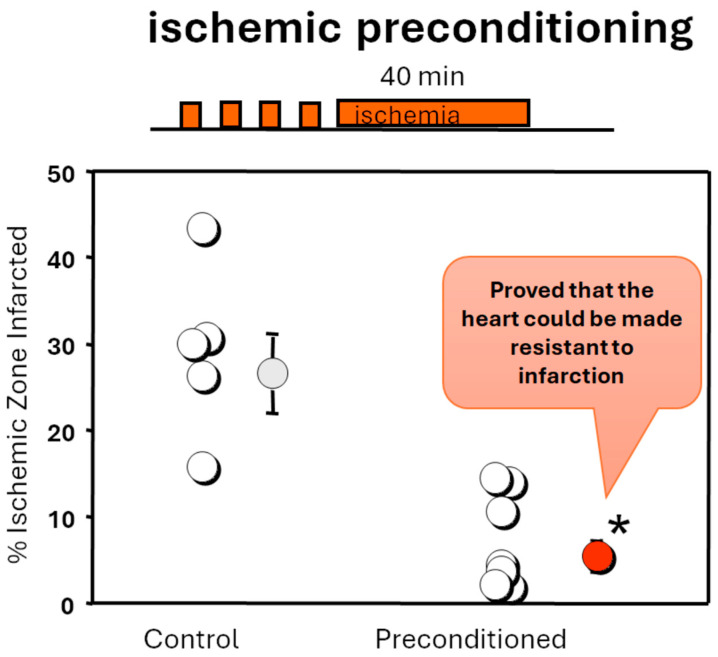
In the seminal study that first described the phenomenon of ischemic preconditioning, the infarct size was measured in open-chest dogs subjected to forty minutes of coronary artery occlusion. In this control group, the infarct size averaged 28% of the risk region. When the index occlusion was preceded by four cycles of five-minute occlusion/five-minute reperfusion, the infarct size was dramatically decreased to an average of 7% of the risk zone. White dots represent myocardial infarct sizes in individual dogs, and gray (control group) and red (experimental group) dots represent group averages. Vertical bars represent standard errors of the mean (SEM). * *p* < 0.001 vs. control. Modified from [21].

But the scientific and clinical worlds were about to be rocked by the most improbable observation. In 1986, Charles Murry, working in the Reimer and Jennings research laboratory, made an unbelievable, but seminal, observation that “more myocardial ischemia was better” [21]. In an open-chest canine model of reversible coronary ligation, a forty-minute coronary occlusion preceded by four cycles of five-minute coronary occlusion/five-minute reperfusion decreased the amount of myocardial necrosis in the anatomical risk zone from 28%, observed in dogs with simply a single transient forty-minute coronary occlusion, to 7% (Figure 1). This 75% reduction in infarction, despite the 50% greater duration of cumulative myocardial ischemia, was initially doubted by others, notwithstanding the respected reputations of the investigators. This phenomenon was labelled ischemic preconditioning (IPC), and several years passed before others attempted to reproduce and further investigate the phenomenon. But once they did, confirmation was widespread and frequent. In fact, the protection of IPC was not just documented in dogs [21,22]. IPC salvaged ischemic myocardium in rodents [23,24], pigs [25], rabbits [26,27], primates [28], and birds [29]. In other words, IPC appeared to be a generalizable phenomenon in all species examined. Clinicians and experimentalists were heartened to now realize that infarction could definitely be modified. But, of course, it was immediately recognized that IPC would have limited clinical utility since the preconditioning ischemia needed to be introduced before the prolonged index coronary occlusion. This would be impossible in clinical settings in which the patient presented with MI after the coronary occlusion and onset of myocardial ischemia. The only possible setting in which IPC might have therapeutic potential would be the operating room during open heart surgery. The dreamed application of the conditioning phenomenon to more routine clinical scenarios would have to wait until its mechanism was better understood.

**Figure 2:**
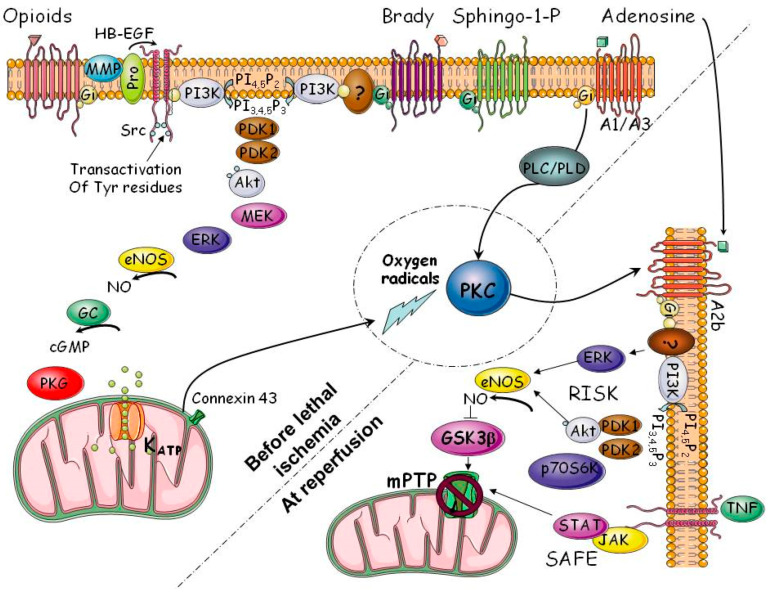
Signal transduction pathway for pre- and postconditioning ischemia and interventions. Abbreviations: Brady = bradykinin, eNOS = endothelial nitric oxide synthase, ERK = extracellular signal-regulated protein kinase, GC = guanylyl cyclase, GSK-3β = glycogen synthase kinase-3β, JAK = Janus kinase, K_ATP_ = ATP-dependent potassium channel, MEK = MAPK kinase, MMP = matrix metalloproteinase, mPTP = mitochondrial permeability transition pore, p70S6K = p70S6 kinase, PDK1/2 = 3′-phosphoinositide-dependent kinase-1/2, PI_3,4,5_P_3_ = phosphatidylinositol trisphosphate, PI3K = phosphoinositide-3-kinase, PKC = protein kinase C, Pro = pro-HB-EGF, RISK = reperfusion injury survival kinases, SAFE = survivor activating factor enhancement, STAT = signal transducer and activator of transcription, TNF = tumor necrosis factor. Reproduced with permission from [31].

We, as well as many others, then spent two decades attempting to define how IPC protected against infarction. The breakthrough came when it was found that the protection involved cellular signal transduction [30]. As depicted in Figure 2, the intracellular signaling sequence of IPC is quite complex [31]. The trigger phase of IPC is initiated by endogenous substances released by ischemic cardiomyocytes: adenosine, opioids, and bradykinin. Whereas adenosine binding to its A_1_ surface receptor leads to the relatively direct activation of protein kinase C (PKC), opioids and bradykinin initiate a much more circuitous activation of PKC. These early signaling steps occur during the ischemic phase of the preconditioning cycle. The transient reperfusion prior to the prolonged index ischemia briefly reintroduces oxygen, which stimulates a burst of free radical production by mitochondrial ATP-sensitive K^+^ channels (mtK_ATP_), leading to the release of reactive oxygen species (ROS) into the cytoplasm and the activation of PKC. PKC activation ushers in the mediator phase occurring during the prolonged index ischemia that ultimately leads to cardioprotection. Signal transduction continues during the mediator phase. We proposed that PKC activation raises the affinity of the adenosine A_2B_ receptor and critically permits the receptor to bind released adenosine at a cellular concentration that otherwise would have been too low for binding [32]. This ability to bind adenosine by A_2B_ receptors appears to be the critical step that separates the preconditioned from the non-preconditioned phenotype. Multiple subsequent signaling steps involving reperfusion injury survival kinases (RISKs) and the alternate survivor activating factor enhancement (SAFE) pathway act to prevent the opening of the mitochondrial permeability transition pore (mPTP), the likely end-effector of IPC. This high conductance pore in the inner mitochondrial membrane dissipates the transmembrane proton/electrochemical gradient that drives ATP generation [33]. An open pore would lead to ATP depletion, increased ROS, membrane ion pump failure, solute entry, and cell swelling and rupture. The acidosis experienced during ischemia inhibits mPTP formation. Reperfusion quickly dissipates the acidosis, and the reintroduction of oxygen increases mitochondrial ROS and calcium entry, leading to mPTP formation. IPC blocks mPTP formation and thus salvages the ischemic myocardium [34,35].

## 5. Ischemic Postconditioning

**Figure 3:**
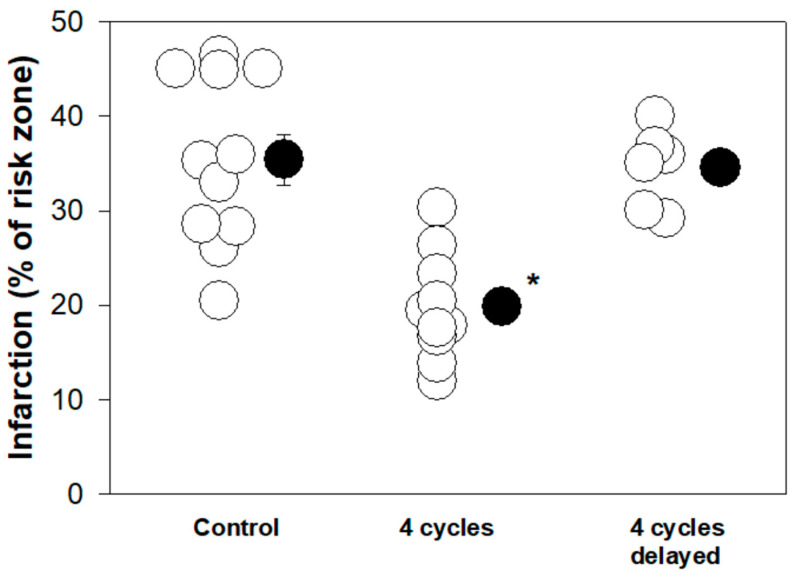
Effect of four cycles of ischemic postconditioning (IPoC) (thirty-second coronary artery reocclusion/thirty-second reperfusion) on the infarct size in open-chest rabbits undergoing thirty-minute coronary artery occlusion/three-hour reperfusion. When IPoC was initiated thirty seconds after the release of the index occlusion, the infarct size averaged 20% of the risk zone, significantly less than the 35% infarction in the control group. However, when IPoC was not initiated until ten minutes after the onset of reperfusion, no protection was observed. White dots represent the infarct size in individual rabbits, and black dots represent group averages with superimposed SEM. * *p* < 0.05 vs. control. Modified from [40].

This roadmap of IPC raised the prospect that an intervention could yield cardioprotection unrelated to the duration of ischemia. Many investigators have actually triggered many of the steps of the IPC signaling pathway directly and mimicked the protection of IPC [31]. But many of the pharmacologic agents could not be used clinically because of toxicity. And there was still the problem that these interventions were being applied before the index coronary ischemia, something not achievable in the clinic. But Hausenloy et al. [36,37] noted that IPC actually exerted its protection at reperfusion. This observation led Vinten-Johansen and colleagues [38] to perform a series of experiments that produced groundbreaking results almost as unbelievable as those reported when IPC was first described. They noted that three thirty-second cycles of reperfusion/reocclusion immediately after the index ischemia and initial reperfusion in dogs were almost as protective as IPC. Others reproduced this cardioprotective intervention, termed ischemic postconditioning (IPoC) [39], and showed that the protection was dependent on the same signals as IPC [40,41], and identified mPTP as the final effector [42,43]. This was the ultimate proof that reperfusion injury contributed to infarction and could be suppressed. Further studies revealed that the success of IPoC required oxygen delivery to the ischemic tissue, while that tissue continued to be acidotic. The continuing acidosis would keep mPTP closed, while reoxygenation activated PKC through redox signaling, leading to the eventual long-lasting prevention of mPTP opening [31]. mPTP inhibition by the activation of the PKC pathway and other signaling steps then persists after the full reoxygenation of the tissue, leading to IPoC’s cardioprotection. The intervals between the brief reperfusion and reocclusion phases of the IPoC protocol must be sufficiently short to prevent pH normalization, while allowing the reintroduction of oxygen to produce ROS and initiate the signaling pathway, leading to the inhibition of mPTP formation [44,45]. As predicted, the reperfusion of the isolated heart after the index ischemia with a low pH buffer in lieu of brief cycles of coronary artery reperfusion/reocclusion mimics IPoC [44,46]. Delaying the application of IPoC for 10 min after the release of the coronary occlusion resulted in a total loss of its protective effect (Figure 3) [40]. A loss of protection was also observed when the delay in instituting IPoC was as brief as one minute [47]. Hence, IPoC must be applied in the first minutes of reperfusion. A delayed IPoC after full tissue reoxygenation and the restoration of the normal tissue pH, which encourages mPTP formation, is no longer cardioprotective. Once mPTP opens, the fate of the ischemic myocardium is sealed, and no additional intervention is likely to be helpful.

Clinical studies can only be performed with agents without toxic side effects, thus excluding many experimental drugs. Amistad I [48] and II [49] evaluated the effectiveness of adenosine in all patients with acute MI (Amistad I) or in those with only high-risk anterior infarcts (Amistad II). The results were disappointing. Atrial natriuretic peptide, which activates PKC in cardiomyocytes [50], had a very modest effect [51]. This modest effect was probably related to the failure to separate low- and high-risk patient groups upon enrollment and, therefore, the dilution of the possible positive results in high-risk patients with the predicted minimal effects expected in lower risk patients. The failure to account for this statistical quirk probably doomed other interventions as well. The Na^+^/H^+^ exchange blockers cariporide [52,53] and eniporide [54], which could have prevented pH normalization during reperfusion and hence mPTP formation, were evaluated despite pre-clinical studies showing efficacy only when the agent was administered before ischemia [55]. The efficacy was minimal, and cariporide increased the risk of strokes. The failure of these expensive clinical trials to demonstrate the efficacy of promising agents based in part on pre-clinical studies made pharmaceutical companies very leery about the likelihood of the successful extrapolation of results from the experimental lab to the clinical theater.

After many years, there was pre-clinical evidence that postconditioning interventions could be therapeutic, but success in the clinic was still being sought. In 2005, in a small study, Staat et al. [56] showed that IPoC with four cycles of one minute of reperfusion followed by one minute of balloon inflation immediately following primary angioplasty in patients with STEMI reduced the rise in total serum creatine kinase by 36% compared to that in patients without IPoC. The expansion of the control and IPoC groups in a subsequent study [57] and again of the control group in a third study [58] demonstrated a 47% decrease in troponin I release in IPoC subjects, and the infarct size by SPECT imaging six months after the procedure was 39% smaller after IPoC. Unfortunately, these results were mostly not confirmed by subsequent rigorous investigations by others [59,60,61,62]. Favaretto et al. [63] performed a meta-analysis of fourteen clinical trials of IPoC in patients being treated for STEMI. Of note, the infarct size was estimated by either enzymes or imaging by cardiac magnetic resonance (CMR). Although the overall analysis revealed a significant reduction in the infarct size, a subsequent evaluation of only the six studies using CMR to directly measure the infarct size revealed no benefit of IPoC.

Cyclosporin, used extensively in organ transplant patients and immune system disorders, directly inhibits the opening of mPTP [64], the end target of pre- and postconditioning. It is cardioprotective in experimental animals [64]. In a small proof-of-concept study, Piot et al. [58], the same group that performed the original IPoC trial [56], documented the effect of cyclosporin administered shortly before primary angioplasty in patients with STEMI. There was a significant decrease in the infarct size in the treated group. These exciting observations prompted these same investigators to initiate CIRCUS, a moderate-sized, multi-center study of intravenous cyclosporin injected shortly before PCI [65]. The primary composite outcome was death from any cause, worsening heart failure during the initial hospitalization, rehospitalization for heart failure, and adverse left ventricular remodeling within one year. The outcomes in the control and treated patients were identical for the composite end-point as well as for each of the four individual components. And peak creatine kinase levels were not different in the two groups. Finally, in the multi-center CYCLE trial, cyclosporin had no effect on the infarct size, left ventricular remodeling up to six months, and clinical outcomes [66].

## 6. Possible Explanation for the Different Results in Pre-Clinical and Clinical Investigations

Pre-clinical studies were so promising! Cardioprotection by either IPC or IPoC was a real phenomenon that had been documented in many species, including primates. Was the human myocardium fundamentally different from the myocardium in laboratory animals? Many possible explanations were offered to account for the negative observations [67,68,69,70,71]. The time of ischemia was much longer in patients than the thirty to sixty minutes often used in animal models. Perhaps clinical study groups were disproportionally weighted, with patients with small infarcts that would have been little affected by a cardioprotective intervention or with patients with very large infarcts that could no longer be salvaged by these interventions. Or possibly partial spontaneous reperfusion prior to PCI made added cardioprotection strategies ineffective. Or the presence of an extensive coronary collateral circulation may have skewed the results. Could ageing, gender, obesity, smoking, and comorbidities, such as diabetes mellitus and hypertension, have influenced the data? Or were the pre-clinical studies not performed with sufficiently rigorous protocols to exclude random effects? Or is the effect of conditioning interventions on the human myocardium really very different from that in all sub-human species? Obviously, it would be extremely difficult to account for all of these confounding variables. But there is one additional confounder. Pre-clinical studies are typically performed by examining the effect of only a single intervention. But patients may be treated with a panoply of agents, such as β-blockers, Ca^++^ channel blockers, nitrates, opioids, ASA, P2Y_12_ antagonists, statins, and anti-diabetic drugs. Although not considered until recently, could any of these drugs have already influenced the data and conclusions of the clinical trials?

## 7. Drugs Administered to STEMI Patients Could Interfere with the Success of Conditioning Interventions

### 7.1. P2Y_12_ Antagonists

**Figure 4:**
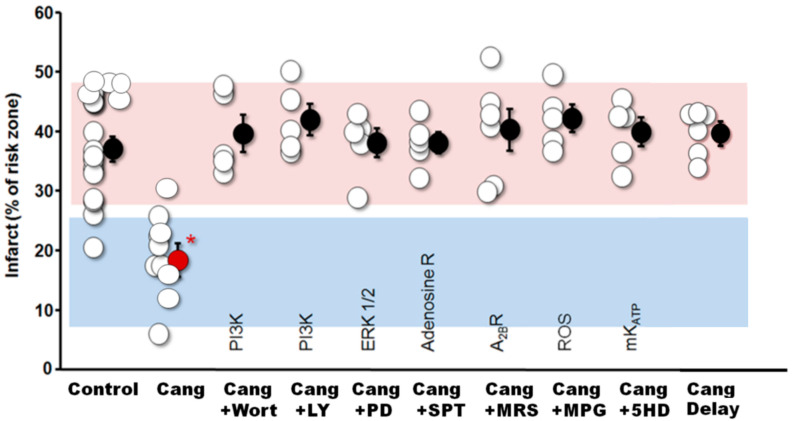
Open-chest rabbits were subjected to thirty minutes of coronary artery occlusion and three hours of reperfusion. When the intravenous infusion of the platelet P2Y_12_ receptor antagonist was started ten minutes before the release of the coronary occlusion, the infarction of the risk region was significantly reduced from 38% in the control group to 19%. However, when the start of the cangrelor infusion was delayed until ten minutes after the release of the thirty-minute coronary occlusion, the infarct size averaged 46%, not different from that in the control group. Cangrelor’s protective effect was eliminated by seven agents known to block the conditioning signal pathway (see Figure 2) when administered minutes before the cangrelor infusion: wortmannin (wort) and LY294002 (LY), blockers of PI3K; PD98059 (PD), a blocker of ERK1/2; 8-sulfophenyltheophylline (SPT), a non-selective antagonist of adenosine receptors; MRS1754 (MRS), a selective antagonist of adenosine A_2B_ receptors; 2-mercaptopropionylglycine (MPG), a scavenger of reactive oxygen species; and 5-hydroxydecanoic acid (5HD), a blocker of mitochondrial K_ATP_ channels. The pink zone represents the range of infarct sizes in unprotected control and treated groups of rabbits, whereas the blue zone represents smaller infarcts consistent with protection. White dots represent infarct size in individual animals, black dots represent group averages with superimposed SEM for unprotected groups, and red dot represents group average for the protected group. * *p* < 0.001 vs. control. Modified from [72].

As already noted, both ASA and P2Y_12_ antagonists are nearly universally employed in patients with STEMI. Their use was predicated on their interference with platelet aggregation and, therefore, thrombus formation. We challenged this assumption in 2012 by examining the effect of cangrelor in a rabbit model of myocardial ischemia/reperfusion [72]. Cangrelor was injected intravenously minutes before the release of a thirty-minute coronary occlusion, which was then followed by three hours of reperfusion. Cangrelor reduced infarction from 38% of the ischemic zone in control animals to 19% (Figure 4). Cangrelor at a dose of 60 µg/kg intravenous bolus followed by 6 µg/kg/min for the duration of reperfusion decreased platelet aggregation by more than 94%. Thus, cangrelor was a very effective blocker of platelet aggregation and was also very effective at blocking reperfusion injury. Was this cause and effect? Of course, ischemia in experimental animals was induced by the mechanical obstruction of a coronary artery and should not have involved thrombus formation, suggesting cangrelor’s protection was not related to its anticoagulant effect. Furthermore, if the cangrelor infusion was delayed by ten minutes following the onset of reperfusion, any cardioprotective effect was abrogated (Figure 4). This observation was identical to that previously seen with IPoC (Figure 3) [40,47]. Could cangrelor be mimicking IPoC? We then did a series of experiments to determine whether cangrelor had any effect on signal transduction. Various signaling molecule antagonists known to block the cardioprotective effects of ischemic or pharmacological postconditioning were administered minutes before the onset of the cangrelor infusion [72]: wortmannin and LY294002, PI3kinase/Akt antagonists; PD98059, an antagonist of MEK 1/2, and, therefore, ERK 1/2; 5-hydroxydecanoic acid, a putative blocker of mtK_ATP_; 8-sulfophenyltheophylline, a non-selective antagonist of adenosine receptors; MRS 1754, a selective antagonist of adenosine A_2B_ receptors; and 2-mercaptopropionylglycine, ROS scavenger and a blocker of redox signaling (see Figure 2). All of these agents aborted cangrelor’s protection (Figure 4). But, importantly, none of the inhibitors affected cangrelor’s profound platelet anti-aggregatory effect. Hence, we were able to divorce cangrelor’s effect on platelet aggregation from its cardioprotective or postconditioning effect. Cangrelor was indeed an authentic conditioning agent, and its protection had little to do with its anti-platelet aggregatory action.

Despite the clear distinction between a cyclopentyltriazolopyrimidine effect on platelet aggregation and an effect on cardioprotection, other data indicate that the protection mediated by P2Y_12_ antagonists is dependent on the presence of platelets. As already noted, cangrelor injected into open-chest laboratory animals just before myocardial reperfusion salvages an ischemic myocardium [72,73,74]. The in vivo murine heart addition of cangrelor just before reperfusion nearly halved infarction [75]. However, unexpectedly, this protection was not observed in the ex vivo, crystalloid-perfused preparation [75]. Nor was cangrelor protective in crystalloid-perfused isolated rabbit hearts [72]. We extended these observations to ticagrelor, and the results were similar [76]. But, notably, isolated, buffer-perfused hearts could still be protected by either IPC [76,77] or IPoC [41]. Therefore, some element carried in the blood was needed to initiate and/or mediate cangrelor’s and ticagrelor’s cardioprotection. We focused on platelets. Rats treated with rabbit anti-rat anti-thrombocyte serum (ATS) were markedly thrombocytopenic after 24 h [78]. Platelet counts fell by 95.4%, while changes in white blood cell count and hematocrit were minimal. Open-chest rats treated with either saline (the control group) or ATS (thrombocytopenic group) had an infarction of approximately 50% of the risk zone. Whereas cangrelor halved infarction after ischemia/reperfusion in the control rats with normal platelet counts, there was no effect in rats made thrombocytopenic with ATS. In contrast, IPC was still quite protective in thrombocytopenic animals [78]. Having established the centrality of the platelet to cyclopentyltriazolopyrimidine’s cardioprotection, we further explored a role for sphingosine [78]. It is known that platelets release sphingosine-1-phosphate (S1P) when activated, and the phosphorylation of platelet membrane sphingosine is indeed an important event in the conditioning pathway (Figure 2) [31,79]. Furthermore, after isolated human platelets are suspended in buffer, exposure to ticagrelor leads to high levels of S1P and low levels of sphingosine in the supernatant compared to the control drug-free preparations [80]. S1P leads to the activation of both the RISK and SAFE pathways [31,79]. N,N-Dimethylsphingosine (DMS) blocks sphingosine phosphorylation by sphingosine kinase [78]. DMS had no independent effect on the infarct size, but completely blocked cangrelor’s cardioprotection [78]. We concluded that P2Y_12_ antagonists are cardioprotective because of the stimulation of the release of sphingosine derivatives from platelets, but their protection is not dependent on any effect on platelet aggregation.

We made an additional observation. In hearts protected by IPoC, the addition of cangrelor had no additional salvage effect [72]. This led us to conclude that the combination of two interventions protecting by the same mechanism are no more effective than either intervention applied singly. But there was still hope that interventions that protected by different mechanisms could still have additive effects.

When the above experiments were performed, the platelet P2Y_12_ antagonist used exclusively in patients with STEMI was clopidogrel. Two days of oral pretreatment of rabbits with clopidogrel was equally as protective as cangrelor, and the protection could be aborted by inhibitors of the conditioning’s signal transduction pathway [72]. Ticagrelor was also cardioprotective [73]. Importantly, cangrelor and IPoC had similar cardioprotective effects in cynomolgous (macaque) monkeys [28,74] and rabbits [72].

These observations may help to explain, at least in part, the disappointing results of the clinical trials described above. In the evaluation by Staat et al. [56] of IPoC in patients with STEMI, the subjects were recruited for the study prior to 2005. In these early years of the twenty-first century, the use of P2Y_12_ antagonists had not yet been universally accepted. Only about half of Staat’s subjects had been premedicated with clopidogrel. Thus, half of the study population could have already been conditioned, which would then have rendered IPoC irrelevant. We urged these investigators to review their database and segregate the patients into control and treatment groups according to clopidogrel usage before the intervention. They did [81]. The control patients already treated with clopidogrel had smaller infarcts than those who were untreated, again supporting the cardioprotective activity of clopidogrel. But, unlike our data in rabbits described above [72], the combination of clopidogrel and IPoC salvaged more tissue than IPoC alone. Could there be an explanation? When we concluded that two interventions with similar mechanisms of action applied simultaneously would have no additional effect beyond that seen with each intervention applied singly, it was understood that each intervention was being studied at a dose or schedule that would have maximized its effect. However, if two submaximal interventions were to be combined, an additive effect would be expected. In experimental animals, dose–response experiments can be performed to determine the optimal protective dose, but this is not possible in humans. Furthermore, a single IPoC sequence based on animal studies was used by the clinical investigators, and it is unknown if shorter or longer brief occlusion or reperfusion times of the repetitive cycles might have been more protective. Additionally, most patients received only 300 mg of clopidogrel, which is clearly suboptimal [82]. Therefore, the suboptimal clopidogrel dosing and uncertainty about the IPoC protocol may have resulted in the application of two interventions that were not maximized, thus resulting in an additive effect.

In the later clinical studies of the effect of either IPoC [59,60,61,62,63] or cyclosporin [65,66], nearly all patients were treated with a P2Y_12_ antagonist, mainly clopidogrel, either in the ambulance or shortly after having arrived at the hospital as dictated by the widely applied society guidelines. In the era of these investigations, PCI could be somewhat delayed for a multiplicity of reasons, thus prolonging the interval between clopidogrel administration and the experimental intervention (IPoC or cyclosporin). Longer intervals would have favored increased clopidogrel absorption and an increased likelihood of a cardioprotective effect. Therefore, the chances of the superimposition of a second conditioning stimulus having a favorable effect were increasingly poor. These considerations emphasize the need for critical pre-clinical investigations in which the effect of an experimental intervention is examined in a clinically relevant animal model, i.e., one in which a P2Y_12_ antagonist has already been administered. Then, it will be possible to determine if the experimental intervention can increase myocardial salvage beyond that already in effect because of the presence of the P2Y_12_ antagonist, currently a standard treatment for all STEMI patients.

Another consequence of the background presence of P2Y_12_ antagonists, which minimizes the effectiveness of other applied postconditioning interventions, is the recognition that, to enhance cardioprotection, a protective strategy not dependent on the conditioning signal transduction pathway (Figure 2) is needed. Then, it will be possible to add the cardioprotective effects of two or more interventions (see below).

### 7.2. ASA

Here is yet another caveat that might shed light on the unexpected modest or absent effect of IPoC and P2Y_12_ antagonists in the clinic. As previously described, ASA is essentially the principal background medication with which all patients with suspected acute MI are treated. Indeed, as soon as patients experience chest pain, they are counseled to consume ASA, and this advice is embedded in all society guidelines. The goal is to inhibit thromboxane A2-dependent platelet aggregation and thrombus formation and propagation. However, recently, the routine use of ASA in STEMI patients has been questioned because of a possible antagonistic effect on potential cardioprotective interventions [83].

Birnbaum and colleagues [84] examined in rats the effect of intravenous ASA infused five minutes before the release of a thirty-minute coronary occlusion. Although ASA itself had no effect on the infarct size, it blocked completely IPoC’s expected myocardial salvage. Additionally, this same group of researchers made similar observations with ticagrelor [85]. Rats were pre-treated with oral ticagrelor and some also received oral ASA by gavage before a thirty-minute coronary occlusion and twenty-four hours of reperfusion. ASA significantly attenuated ticagrelor’s protective effect. When rats pre-treated with oral ticagrelor also received an injection of a specific COX2 inhibitor minutes before coronary occlusion, ticagrelor’s protection was completely aborted. This interference with postconditioning by ASA could also have contributed to the failure of the IPoC and cyclosporin clinical trials (all subjects were treated with ASA before PCI).

Several clinical studies supply suggestive information about the interaction of P2Y_12_ antagonists and ASA. In the ATLANTIC study, all subjects were treated with ASA. The effect on the S-T segment elevation of oral ticagrelor administered in the ambulance to STEMI patients was compared to ticagrelor’s effect in a group in which it was orally administered just before PCI [86]. In the latter group, ticagrelor absorption leading to effective platelet P2Y_12_ antagonism did not occur for at least one hour and probably longer after PCI. Since the salvage effect of P2Y_12_ antagonists in reperfusion injury is absent if the agent is administered beyond the early minutes of reperfusion [72], this second clinical group can be considered to be the putative control group that was effectively exposed to only ASA during those early critical moments of reperfusion. Based on pre-clinical observations, one might have expected less ischemia and a faster resolution of S-T segment elevation in the ticagrelor early treatment group. However, there was no difference between the two groups. It is appreciated that the median time of ticagrelor loading between the two groups was only thirty-one minutes, possibly accounting for the absence of any difference in the resolution of S-T segment elevation or the rate of major adverse cardiovascular events. But it should not be overlooked that all patients were treated with ASA, which might have interfered with a potentially positive ticagrelor effect.

Additionally, Ubaid et al. [87] evaluated the effect of cangrelor administered a few minutes before PCI in a group of STEMI patients who were also treated with oral ticagrelor after PCI and 1.5 h after the initiation of the cangrelor infusion. The infarct size assessed by CMR three months later in this group was compared to that measured in a comparable clinical group without cangrelor administration but with oral ticagrelor loading after randomization and before PCI. At the time of PCI, platelet aggregation was greatly depressed in the cangrelor group, signifying the expected anti-platelet P2Y_12_ effect, but hardly noticeable in the ticagrelor group, which can then be considered to be a functional control group effectively exposed to only ASA at the time of PCI. Troponin levels at 24 h and the infarct size by CMR at three months were equivalent in the two groups. Based on pre-clinical data cited above, the cangrelor group should have had smaller infarcts. Again, all patients were treated with ASA. Hence, a direct inhibitory effect of ASA cannot be proved, but it is possible that ASA blocked any salutary effect that might have resulted from the cangrelor treatment. We suggest, again, that before any cardioprotective strategy is assessed in a clinical trial, it should be evaluated in a pre-clinical model in which both the control and treatment groups include pharmacologic agents that mimic the clinical scenario as closely as possible.

## 8. Multiple Simultaneous Interventions

**Figure 5:**
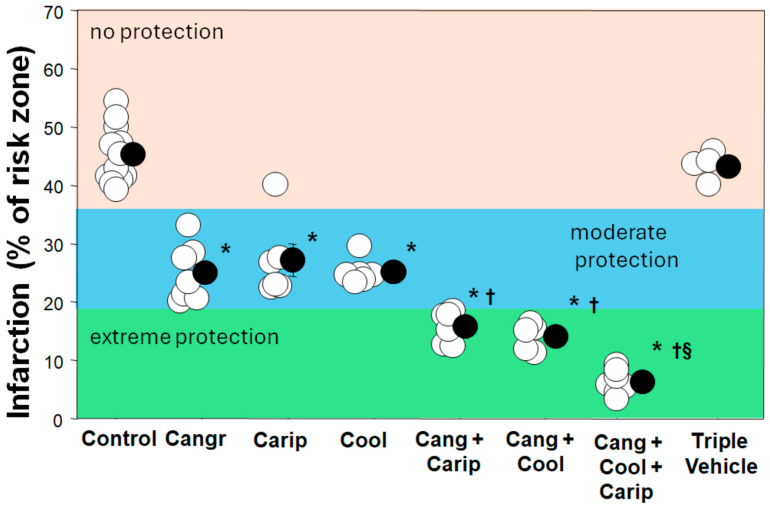
Infarct size in open-chest rats subjected to thirty minutes of coronary artery occlusion and two hours of reperfusion was significantly decreased from 45% of the risk region in the control group to approximately 25% by either cangrelor infused ten minutes before reperfusion, cariporide administered before the coronary occlusion, or cooling commenced ten minutes before occlusion and resulting in body temperature of 32–33 °C by the start of occlusion. When the interventions were combined in pairs (cangrelor + cariporide or cangrelor + cooling), the infarct size was further decreased to approximately 14–16%. And when all three interventions were combined, infarction fell further to 6%. The pink zone represents the range of infarct sizes signifying no protection; the blue zone represents the range of infarct sizes signifying moderate protection; and the green zone represents the range of infarct sizes signifying marked protection. White dots indicate infarct size in individual rats, while black dots indicate group averages. * *p* < 0.001 vs. control; ^†^ *p* < 0.001 vs. Cangrelor or Cariporide or Cooling; ^§^ *p* < 0.001 vs. Cangrelor + Cariporide or Cangrelor + Cooling. Modified from [73].

**Figure 6:**
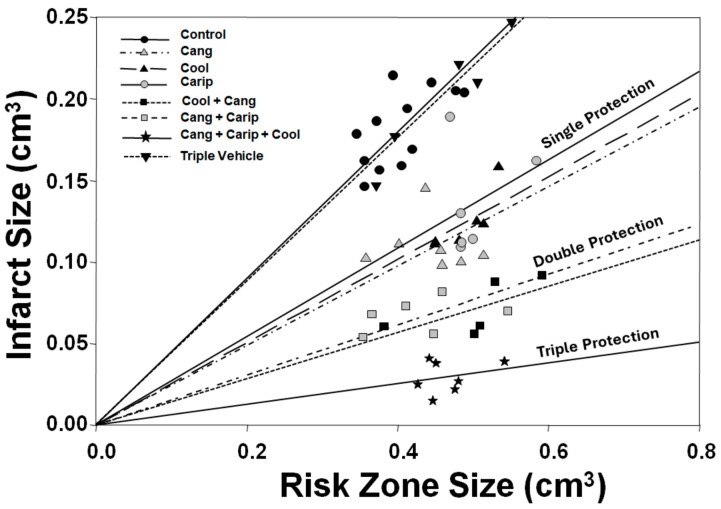
Infarct size in the same rats described in Figure 5 plotted against the risk zone size for the control animals without intervention, rats treated with individual interventions (cangrelor, cariporide, and cooling), rats treated with doubled interventions (cooling + cangrelor and cangrelor + cariporide), and rats treated simultaneously with all three interventions. The lines are the least squares fit through the origin. For any risk zone size, the infarct size is progressively lowered as the number of simultaneous interventions is increased. Modified from [73].

It should be noted that none of the interventions that clearly reduce the infarct size in animal models actually abolished infarction. So, what is killing the cells that the cardioprotectant missed? Does this mean that multiple factors independently contribute to infarction? If so, another cardioprotectant that targets an additional factor should provide additive protection. We have previously discussed interventions that protect by the same mechanisms, such as cangrelor and IPoC. We examined several interventions that had distinctive cardioprotective profiles to determine if additive cardioprotection could be achieved [73]. In an instructive experimental study, we evaluated cangrelor, the prototypical postconditioning intervention, when added to IPC, the Na^+^/H^+^ exchange blocker cariporide, or mild hypothermia in open-chest rats. As expected, a cangrelor bolus and then continuous infusion, started ten minutes prior to the end of a thirty-minute coronary occlusion, decreased the infarct size from 45.3% of the risk zone in untreated hearts to 25.0% (Figure 5). As expected, the addition of IPC to cangrelor-treated hearts had no effect. Conversely, peritoneal lavage with cold saline just prior to coronary occlusion, which lowered the body temperature to 32–33 °C, decreased infarction to 25.2% of the risk zone. Cariporide lowered infarction to 27.2%. Therefore, all single interventions were equipotent and approximately halved infarction (Figure 5). The combination of cangrelor and hypothermia further lowered infarction to 14.1%, and infarction in rats treated simultaneously with cangrelor and cariporide was 15.8% of the risk zone (Figure 5). Finally, the combination of cangrelor, hypothermia, and cariporide additionally halved infarction to 6.3% (Figure 5). The increasing power of the protective effect as interventions were first grouped in pairs and then as a combined triple treatment is evident when the infarct size is plotted against the risk zone (Figure 6). For any given risk zone size, the infarct size is much smaller when hearts are exposed to single cardioprotective interventions, smaller again when two interventions are paired, and almost negligible when all three interventions are combined. Therefore, it is indeed possible to combine interventions and produce additive protective effects. It may well be that any single intervention produces protection that is too modest to be detected in the clinical setting, in which a wide range of infarct sizes is observed in each group. There are clearly multiple processes that contribute to ischemia/reperfusion-induced cell death. Three were identified above: high temperature during ischemia, the opening of mPTP, and calcium entry and resolution of acidosis after reperfusion. Are there more? In a recent review, seven different processes were described that appear to contribute to cell killing during ischemia/reperfusion [88]. How many can be targeted to offer additive protection? Will the search for interventions that can be combined become the next frontier of investigation? We identified one candidate that could influence infarction: inflammation.

## 9. Inflammation Contributes to Reperfusion Injury

Inflammation has been considered to be an important etiologic factor in vascular and myocardial disease by some for over 100 years [89]. However, for most, the evidence was unconvincing until the insightful investigations of Libby and colleagues established that inflammation was indeed intimately linked to the atherosclerotic process [90], and was also responsible for the destabilization of vascular plaques, coronary thrombosis, and myocardial infarction [3,4]. In the pathobiology of myocardial infarction, it was established that leukocytes, including mast cells and lymphocytes, express proinflammatory cytokines and promote production by atherosclerotic plaques of proteases, procoagulation factors, and inflammatory cytokines, which in turn destabilize plaques and modulate thrombus formation. Once the inflammatory process was generally accepted as a critical factor in the genesis, growth, and eventual rupture of coronary plaques, the focus turned to identifying acceptable interventions that would interfere with this inflammation. Because inflammation and leukocytes are involved in tissue repair after an injury, it was feared that attempts to tamp down inflammation to prevent plaque rupture and myocardial infarction would interfere with healing, resulting in poor scar integrity and thinned fibrotic patches replacing the necrotic myocardium. In fact, this was observed when steroids were used to decrease inflammation after myocardial infarction [91]. It behooved investigators to select more targeted anti-inflammatory interventions.

In the last ten years, several clinical studies have been completed that support the concept that selected anti-inflammatory agents can have a positive impact on the clinical course of ischemic heart disease. Colchicine is an anti-inflammatory drug that has been used for a variety of inflammatory ailments, including gout and other arthritic afflictions and pericarditis. It inhibits microtubule assembly in cells and is a non-specific inhibitor of the NOD-, LRR-, and pyrin domain-containing protein 3 (NLRP3) inflammasome by blocking component assembly and diminishing the macrophage secretion of inflammatory proteins [92]. In the LoDoCo [93] and COLCOT [94] investigations in individuals with chronic, stable coronary artery disease or recent MI, colchicine significantly decreased a combination of cardiovascular death, stroke, acute coronary syndrome, and cardiac arrest. However, in the Australian COPS investigation in patients recovering from an acute coronary event, colchicine had no effect on cardiovascular outcomes at 12 months [95].

Several more specific treatments have generated great interest. In the Cantos study, canakinumab, a human monoclonal antibody targeting interleukin (IL)-1β, in patients with known coronary heart disease and evidence of systemic inflammation (elevated high-sensitivity C-reactive protein) significantly decreased the composite end-point of MI, stroke, and cardiovascular death as well as heart failure [96,97]. Abbate and colleagues [98] and Morton et al. [99] studied the effects of two weeks of anakinra, a recombinant IL-1 receptor antagonist, in patients recovering from MI, many treated with PCI. In the immediate post-MI period, there was no effect of treatment on troponin release. Three and twelve months later, the frequency of ischemic events was not different in the control and treated subjects, although in Abbate’s study, the frequency of new heart failure was decreased in the subjects who had been treated with anakinra.

These clinical studies were not designed to study the effect of anti-inflammatory agents on reperfusion injury since the agents were administered hours, days, or longer after the clinical ischemic event. Rather, these protocols evaluated the effect of anti-inflammatory agents on post-ischemic remodeling. And the results were mixed. Only Broch et al. [100] evaluated an intervention applied during early reperfusion. They injected tocilizumab, an IL-6 inhibitor, during PCI. The infarct size was evaluated three to seven days later with CMR. The treated subjects had larger myocardial salvage indices (69% vs. 64%, *p* = 0.04). Although the absolute infarct size was lower in the treated subjects at both three to seven days and six months after treatment than in the untreated subjects, the differences were not significant.

Studies in STEMI subjects provide both encouraging and negative data about the effects of anti-inflammatory agents in ischemic heart disease. Of course, it is possible that the tested agents were not the correct ones to test. Certainly, the timing of the administration of the experimental drugs was not appropriate if the goal was to attenuate reperfusion injury. Perhaps, a better appreciation of the inflammatory process would help in the selection of better agents.

**Figure 7:**
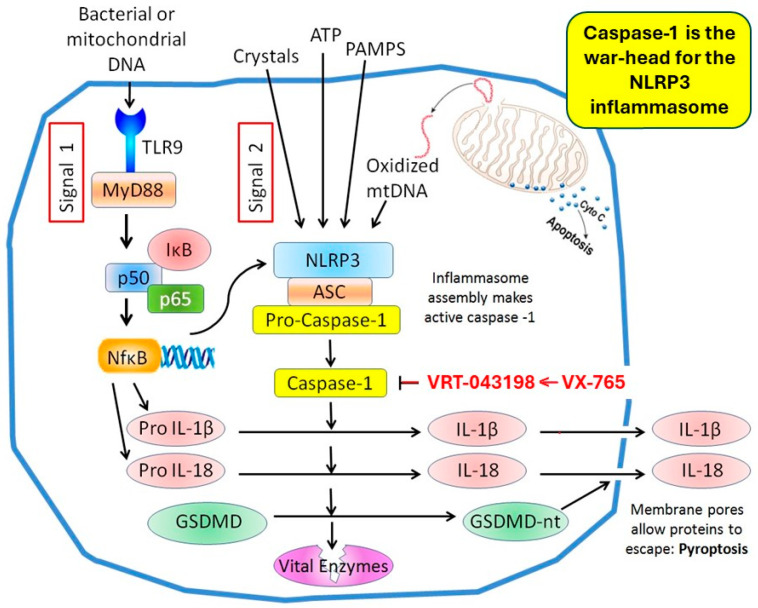
Diagram of the NLRP3 inflammasome, its priming and activation sequences, and its component proteins. Caspase-1 is the killer protease that cleaves pro-cytokines into active IL-1β and IL-18 and also gasdermin D into its C-terminal and N-terminal moieties. The latter binds to the lipids of the inner plasma membrane and forms pores allowing the cellular contents to escape and solutes and ions to enter the cell, leading to cell swelling and rupture, a process termed pyroptosis. The pro-drug VX-765 is converted by blood-borne esterases to its active form, VRT-043198, a potent caspase-1 inhibitor. Abbreviations: ASC = apoptosis-associated speck-like protein, Cyto C = cytochromne C, GSDMD = gasdermin D, GSDMD-nt = GSDMD amino-terminal cell death domain, IKB = inhibitor of KB, mtDNA = mitochondrial DNA, MyD88 = myeloid differentiation primary response 88, NF-κB = nuclear factor-κB, NLRP3 = NOD-, LRR- (leucine-rich repeat-) and pyrin domain-containing protein 3, PAMPs = pathogen-associated molecular patterns, TLR = Toll-like receptor. Modified from [88].

The NLRP3 inflammasome is central to an organism’s anti-inflammatory response to viral and bacterial invaders and foreign proteins. The appearance of one of the latter initiates complex priming and activation sequences [101,102,103,104]. The NLRP3 inflammasome is made up of three major components (Figure 7): the NLRP3 sensor, the apoptosis-associated speck-like protein (ASC) adaptor, and the caspase-1 (cas-1) effector [101,105]. NLRP3 itself consists of three proteins: an amino-terminal pyrin domain (PYD), a central NACHT domain (consisting of four sub-domains: NAIP, CIITA, HET-E, and TP1), and a carboxy-terminal leucine-rich repeat (LRR) domain [101,105]. The latter naturally folds back onto the NACHT domain, resulting in autoinhibition. ASC has an amino-terminal PYD domain and a carboxy-terminal cas recruitment domain (CARD) [101,105]. The amino-terminal CARD, a large, central catalytic (p20) domain, and a carboxy-terminal (p10) domain make up pro-cas-1 [105,106]. When stimulated, NLRP3 oligomerizes and recruits ASC [101,105]. Multiple ASC filaments coalesce into an ASC speck, which recruits pro-cas-1 through CARD-CARD interactions [101]. Cas-1 can then self-cleave to become enzymatically active [105,106].

## 10. Gasdermin D and Caspase-1 Play Pivotal Roles in the Pyroptosis-Induced Death of Cardiomyocytes

As already noted, the generation of a biologically active inflammasome is a two-step process. This grand ballet of interrelated protein transformations and translocations begins with the recognition of pathogen-associated molecular patterns (PAMPs) or damage-associated molecular patterns (DAMPs) by pattern recognition receptors, leading to priming (Figure 7) [101,102,103,104].

The second phase of the process results in inflammasome formation from its individual components and activation. A diverse variety of stimuli, e.g., bacterial, viral, or fungal PAMP or any one of a variety of DAMPs including crystals, β-amyloid, oxidized mtDNA, and sphingosine, can stimulate inflammasome assembly [101]. Because there are so many diverse stimuli, it is unlikely there is a direct interaction between NLRP3 and these agonists. Rather, they are presumed to induce some form of cellular stress, which is sensed and, in turn, leads to upstream signals, such as K^+^ efflux, Ca^++^ influx, mitochondrial ROS generation, and oxidized mtDNA formation, which can activate the NLRP3 inflammasome [101,103,107]. During the sequence of activation, NLRP3 relocates from the endoplasmic reticulum to the perinuclear space, while ASC moves there from mitochondria, and it is here that these two components interact [108]. The NLRP3 PYD domain recruits the PYD of ASC [103,105]. The latter’s CARD, in turn, recruits the CARD of pro-cas-1 [103,105]. Cas-1 is a cysteine protease. After the recruitment of pro-cas-1 to the inflammasome platform, pro-cas-1 dimerizes followed by autoproteolytic cleavage. A conformational change results in the proximity of the two active sites forming a functional catalytic site [105,106].

Activated cas-1 is a potent protease that targets several intracellular proteins, resulting in profound biologic consequences. During the priming process before NLRP3 assembly and activation, pro-IL-1β and pro-IL-18, cas-1 substrates, are upregulated [101,103]. Cas-1 cleaves pro-IL-1β at an aspartic acid site, resulting in a mature proinflammatory cytokine IL-1β, central to the cell’s response to infection [106]. The primary route for IL-1β leakage is an explosive release through a permeabilized cell membrane caused by pore formation mediated by another cas-1 cleavage protein, gasdermin D (GSDMD) [109]. IL-18 undergoes the same processing as IL-1β.

GSDMD is one of a family of proteins originally identified in the skin and the G-I tract [110]. It is found in immune cells, at mucosal sites, and in many of the body’s tissues. The unstimulated protein contains a C-terminal domain that folds back onto the N-terminal domain, resulting in autoinhibition. Cas-1 activates GSDMD by the cleavage of its interdomain linker region at an aspartate residue releasing the N-terminal domain, which oligomerizes, translocates to the inner plasma membrane, and binds to lipids and phosphoinositides [101,111]. A multi-unit barrel pore with a diameter approximating 20 nm is formed [110,111]. These pores act as channels through which the cellular contents, including proteins and IL-1 cytokines, are released into the extracellular space. Solutes and ions pass through the channels into the cell resulting in cell swelling, membrane rupture resulting in the explosive release of cell contents, and cell death. This process of programed cell death is termed pyroptosis.

Cas-1 is obviously a critical component of the inflammasome, and the upregulation of its activity initiates both the salutary generation of inflammatory cytokines critical to the establishment of an effective response to invading pathogens and the destructive cleavage of GSDMD, leading to pore formation in the sarcolemma and cell death. Cas-1 activation must be carefully controlled to prevent inadvertent cell damage. As described below, cas-1 activation appears to be localized to the reperfusion phase of an ischemia/reperfusion insult. What type of stimulus at reperfusion might accelerate cas-1 activation? Ca^++^ floods the ischemic cell at the onset of reperfusion, and Ca^++^ has often been implicated as a cause of reperfusion injury. As already noted, Ca^++^ influx through membrane ion channels stimulates inflammasome assembly [101,103,107]. Ca^++^-activated calpain can also activate cas-1. Calpains are cysteine proteases and, in their inactive state, exist as heterodimers with large catalytic and small regulatory subunits [112,113]. The low pH of ischemic cells renders calpain inactive [114,115]. Calpain activation by Ca^++^ during reperfusion occurs as pH rapidly increases. Ca^++^/calmodulin-dependent protein kinase II (CaMKII) in the cytosol binds Ca^++^ and autophosphorylates and then binds calpain [113]. CaMKII carries its substrate calpain to the sarcolemmal membrane, where, in the presence of phospholipids, calpain is likely phosphorylated and cleaved to its active catalytic form [113]. Calpain translocation occurs during myocardial ischemia, whereas Ca^++^ binding and activation happen only after the onset of reperfusion and pH normalization. Calpain frees pro-cas-1, which is normally bound to cytoskeletal actin by flightless-1 protein [116]. Once released into the cytosol, pro-cas-1 can become part of the assembling NLRP3 inflammasome or can autocleave into enzymatically active cas-1.

The signaling involved in inflammasome activation and pyroptosis is complex. There are multiple steps that have been scrutinized to determine if blockade or downregulation might influence the course of MI. Many investigators have confirmed that myocardium contains the inflammasome machinery. This is not surprising since there are resident cardiac fibroblasts and macrophages. But inflammasomes are also found in cardiomyocytes themselves [117,118,119,120,121]. Indeed, the hypoxia/reoxygenation of rat cardiomyocytes leads to the release of the N-terminal fragment of GSDMD and cas-1, while their release is significantly curtailed when NLRP3 is inhibited [122]. Furthermore, after NLRP3 inhibition, the expected loss of membrane integrity and the release of LDH as a marker of membrane failure are no longer apparent.

In an isolated rat heart subjected to ischemia/reperfusion, myocardial NLRP3 and cas-1 levels increased three-fold and the IL-1β content doubled [123]. Interestingly, the tissue GSDMD level was decreased by 30% after sixty minutes of reperfusion, while that of the GSDMD N-terminal subunit was increased six-fold in the untreated heart. INF-4E, an NLRP3 inhibitor, nearly halved the protein level of NLRP3 after sixty minutes of reperfusion, modestly but significantly decreased pro-cas-1 cleavage, and caused small, significant decreases in GSDMD cleavage. Curiously, INF-4E pretreatment increased the phosphorylation of ERK, Akt, and glycogen synthase-3β (GSK-3β), indicating certain similarities to the protective mechanism of IPC (Figure 2) (see below).

Darwesh et al. [124] evaluated isolated murine heart tissue following ischemia/reperfusion. The tissue NLRP3 level increased 4-fold, the IL-1β content increased 2.5-fold, and cas-1 activity increased 4-fold. There can be little argument that ischemia/reperfusion dramatically increases pyroptosis-associated molecular components. And these measurements were made in buffer-perfused hearts, thus excluding any contribution from invading leukocytes. On the basis of these biochemical investigations, ischemia/reperfusion results in predictable changes in the amounts of the intracellular components of the NLRP3 inflammasome. And these changes were aborted when inflammasome activation was pharmacologically blocked. How did the attenuation of inflammasome activation affect myocardial integrity?

Pomerantz and colleagues [125] exposed human atrial muscle segments to hypoxia/reoxygenation and measured the contractile force. The expected loss of contractility after hypoxia/reoxygenation was attenuated by the blockade of IL-1 receptors or cas-1. Mastrocola et al. [123] measured the infarct size in isolated rat hearts after thirty minutes of ischemia and sixty minutes of reperfusion. The pretreatment of the hearts with INF-4E resulted in small, but significant decreases in the infarct size. Kawaguchi and colleagues [126] inhibited NLRP3 inflammasome formation by knocking out ASC in murine hearts, which were then subjected to thirty minutes of regional coronary artery ischemia and two days of reperfusion. The infarcts were smaller in ASC^-/-^ than in wild-type mice. The same was evident in cas-1^-/-^ mice.

Marchetti et al. [127] evaluated murine hearts exposed to thirty minutes of coronary artery occlusion and twenty-four hours of reperfusion. The pretreatment with the NLRP3 inflammasome formation inhibitor 16673-34-0 decreased the infarct size by 40% and inhibited cardiac cas-1 activation by approximately 90%. In a subsequent study, 16673-34-0 administered to mice at reperfusion after thirty minutes of myocardial ischemia reduced the infarct size by 56% and improved the cardiac contractility [128]. Thus, the injury occurred sometime during reperfusion. Using the same model, these investigators made novel conclusions about the temporal effect of inflammasome blockade on reperfusion injury [129]. The infarct sizes in untreated mice after one, three, and twenty-four hours of reperfusion were 11%, 30%, and 43%, respectively. The injection of the NLRP3 inhibitor at the start or one hour after the onset of reperfusion decreased both cas-1 activity and the infarct size after twenty-four hours. On the other hand, neither cas-1 activity nor the infarct size were affected when the antagonist was administered after three hours of reperfusion. Therefore, these data suggest that NLRP3 inflammasomes exert their injurious effect on the reperfused myocardium sometime during the first three hours of reperfusion.

In pigs treated with seventy-five minutes of coronary occlusion and seven days of reperfusion, the NLRP3 inflammasome inhibitor MCC950 reduced the infarct size by 17% [130]. We noted a similar effect of MCC950 in isolated mouse hearts after fifty minutes of ischemia and two hours of reperfusion [131]. BAY 11-7082 is another NLRP3 inhibitor. When administered prior to the onset of reperfusion in rats, the infarct size was decreased by approximately 20% [132]. The increased myocardial NLRP3, ASC, cas-1, and IL-1β levels noted in untreated ischemic myocardial tissue were attenuated by BAY 11-7082.

In contrast to van Hout’s earlier study with MCC950 [130], this same group was unable to demonstrate any effect of a different NLRP3 inhibitor on the infarct size in pigs undergoing seventy-five minutes of balloon occlusion of the left anterior descending coronary artery followed by seven days of reperfusion [133]. IZD334 successfully decreased IL-1β release from porcine peripheral blood mononuclear cells after treatment with lipopolysaccharide or ATP. But it had no effect on the infarct size when administered intravenously fifteen minutes before reperfusion and orally for the next six days. IL-1β levels were equally low in the myocardial tissues of control and treated hearts when harvested after seven days of reperfusion.

Several studies with genetically altered animal models have not confirmed the results of the majority of infarct studies described above. Sandanger and colleagues [134] evaluated isolated, perfused murine hearts from NLRP3^-/-^- and ASC^-/-^-deficient animals. An improved post-infarction contractility and decreased infarct size were observed in NLRP3^-/-^ but not ASC^-/-^ hearts. This mixed result was further explored in in situ mouse hearts [135]. The investigators found that the infarcts were actually larger in NLRP3^-/-^ hearts. This unexpected result might be related to the overexpression of TLR4 in NLRP3^-/-^ hearts [136] or possibly another unidentified phenotype alteration in these knockout animals.

Jong et al. [137] observed that IL-1β levels were similar in the risk area of wild-type and NLRP3^-/-^ mice after coronary artery occlusion and three hours of reperfusion. Additionally, waiting 10 days after exteriorizing a coronary artery ligature occluder to allow the effects of the surgical trauma to dissipate before creating a thirty-minute coronary artery occlusion and then three hours of reperfusion resulted in no difference in the infarct size in wild-type and NLRP3^-/-^ mice, and no NLRP3 protein was detectable in the heart’s risk region. When the same ischemia/reperfusion protocol was used in an open-chest animal model, NLRP3 was detected in hearts of wild-type animals but obviously not in knockout mice. The authors concluded that the increased post-ischemia/reperfusion NLRP3 in myocardial risk zones measured by other investigators was the result of inflammation related to the surgical procedure, and, therefore, artifactual. Of course, this is not compatible with the observations of others who have measured NLRP3 and other components in buffer-perfused, isolated heart models without circulating blood cells.

The above scientific investigations have principally examined effects of the genetic elimination of NLRP3 or the latter’s pharmacologic antagonism on the cytoplasmic levels of inflammasome components, the release of downstream inflammatory cytokines and other intracellular proteins, and myocardial contractility and infarct size following ischemia/reperfusion. The preponderance of evidence supports the direct deleterious effect of inflammation on myocardial integrity. These data are bolstered by interventions blocking the activation of the downstream killer protease, cas-1.

Pomerantz et al. [125] studied the effect of the cas-1 inhibitor YVAD in human atrial muscle strips exposed to hypoxia/reoxygenation. YVAD improved post-ischemic contractility. As previously noted, Kawaguchi and colleagues [126] observed that the infarct size in a murine model of ischemia/reperfusion was smaller than that in the cas-1^-/-^ knockout group.

## 11. Caspase-1 Blockers

While many studies have focused on the suppression of the NLRP3 inflammasome with pharmacologic inhibitors, we reasoned that cas-1 might be a simpler and more effective target for the prevention of pyroptosis in the reperfused heart. Small-molecule cas-1 inhibitors have been developed [138]. The one that we have favored is VX-765, which acts through the reversible covalent modification of cas-1′s catalytic cysteine residue [139]. We selected VX-765 because it had been used in several clinical trials for the treatment of dermatological and neurologic ailments [140,141,142]. VX-765 is an oral preparation, but it is not an active molecule. When the pro-drug VX-765 is absorbed from the gut into the bloodstream, it is cleaved by circulating esterases to yield the active component VRT-043198 [139]. Because of the requirement of modification by esterases, VRT-043198, not VX-765, injected at the beginning of reperfusion, is protective in buffer-perfused, isolated heart preparations that are esterase-deficient [143]. VRT-043198 blocks the liposaccharide-stimulated IL-1β and IL-18 release from human peripheral blood mononuclear cells, and oral VX-765 in mice decreases the cytokine response to lipopolysaccharide by up to 60% [139].

In our first study of VX-765 open-chest rats were treated intravenously with VX-765 thirty minutes prior to the onset of one hour of ischemia followed by two hours of reperfusion [144]. In untreated rats, the infarct size averaged 74% of the risk zone. VX-765 nearly halved the infarct size. Do Carmo and colleagues [145] observed similar results. But, of course, in these experiments, VX-765 was administered before ischemia, and, therefore, it was not possible to assume it was targeting reperfusion injury. We modified the experiments by administering a single intravenous bolus of VX-765 five minutes prior to two hours of reperfusion after sixty minutes of coronary artery occlusion [143]. VX-765 decreased infarction from 60% of the risk zone to an average of 29%. The serum level of IL-1β was decreased. Chest closure and animal sacrifice after seventy-two hours of reperfusion showed that infarcts were no larger than those observed after two hours of reperfusion, implying VX-765 had protected the reperfused heart early after reperfusion with no additional infarction after drug wash-out. Since the pretreatment of rats with VX-765 resulted in infarcts no smaller than those seen when VX-765 was injected five minutes before reperfusion, it was plausible to conclude that all cas-1-dependent myocardial damage occurs at reperfusion in this animal model.

**Figure 8:**
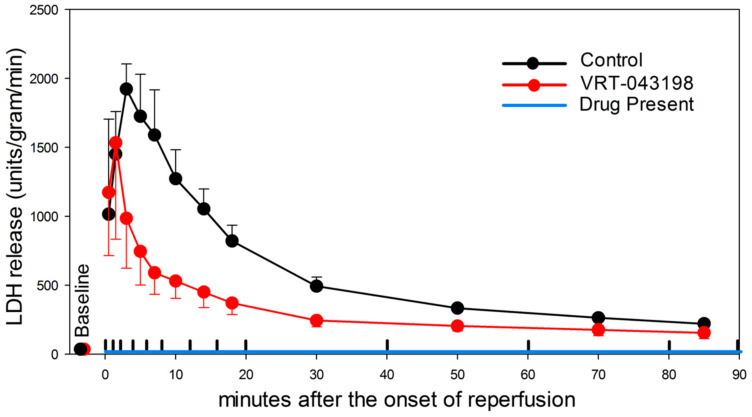
The effluent was collected at timed intervals from isolated rat hearts perfused with Krebs buffer during reperfusion following forty minutes of global ischemia. An aliquot of each sample was assayed for LDH, which was released into the effluent following cell rupture. Therefore, it is a marker of pyroptosis. The black dots represent measurements in control hearts without intervention, while the red dots represent measurements in hearts treated with the caspase-1 antagonist VRT-043198 added to the aortic perfusate throughout reperfusion. LDH release into the effluent in the untreated control hearts peaked at five minutes and diminished steadily thereafter. Notably, in the hearts treated with VRT-043198, the LDH release peaked at two minutes and was significantly diminished throughout the reperfusion compared to the untreated hearts. Therefore, caspase-1-dependent injury occurs very early during reperfusion and is significantly attenuated by blockade of the protease. The areas under the curves for the control and VRT-treated hearts are significantly different. Reproduced with permission from [143].

The timing of physiologically relevant cas-1 blockade is obviously an important issue, especially if one hopes to extrapolate the use of cas-1 blockers to the treatment of STEMI. The effluent from isolated hearts treated with VRT-043198 at reperfusion was collected and analyzed for LDH, a marker of membrane failure in pyroptosis [143]. In untreated hearts, LDH was released into the effluent, peaked within five minutes of reperfusion and decreased thereafter (Figure 8). In contrast, the infusion of VRT-043198 at reperfusion significantly decreased LDH release during the entire reperfusion period. Therefore, cas-1-dependent injury occurs early during reperfusion. As already described, the data of Toldo et al. [129] suggested treatment with an NLRP3 antagonist could be delayed for at least one hour without the loss of a salutary effect on reperfusion injury, but not for three hours. In contrast, our prior observations with platelet P2Y_12_ antagonists [72] and IPoC [40,47] had demonstrated that the administration of the drug or the repetitive postconditioning cycles of ischemia and reperfusion ten minutes after the onset of reperfusion resulted in the loss of protection from the anti-platelet inhibitor or IPoC. VRT-043198 infused at reperfusion in isolated mice hearts after a fifty-minute coronary occlusion decreased infarction from approximately 90% of the risk zone to approximately 45% [146]. But, when the perfusion of the heart with VRT-043198 was delayed until ten minutes after the onset of reperfusion, the protection was lost. These data continue to confirm that an effective treatment must be applied in the early minutes of reperfusion if reperfusion injury is to be diminished.

## 12. Calpain

In isolated murine cardiomyocytes subjected to hypoxia/reoxygenation, calpain small interfering RNA increased the survival rate of cardiomyocytes while inhibiting the usual increase in NLRP3, ASC, and cas-1 [147]. Furthermore, blocking Ca^++^ entry into ischemic cells with a Na^+^/Ca^++^ exchange inhibitor, which effectively attenuates calpain activation, also diminished LDH release from isolated rat hearts after ischemia/reperfusion [114]. In both isolated heart and open-chest models in rodents, rabbits, and pigs, infarction after ischemia/reperfusion is decreased by multiple pharmacologic calpain inhibitors [112,114,115]. We evaluated the calpain inhibitor calpeptin in isolated murine hearts [146]. Whereas infarction in control hearts averaged approximately 90% of the risk zone after fifty minutes of global ischemia followed by two hours of reperfusion, calpeptin administered initially before ischemia and continued until the end of reperfusion decreased infarction to approximately 60%. This degree of protection was similar to that seen with IPC as well as the pan-cas inhibitor emricasan [146], which is chemically distinct from VX-765 and which has also previously been used in clinical trials [148].

## 13. Gasdermin D Antagonism or Genetic Modification

Recently, Zhong et al. [149] developed a potent inhibitor of GSDMD. GI-Y1 binds to GSDMD and inhibits the lipid-binding and pore formation of the N-terminal cleavage moiety. Mice hearts were exposed to thirty minutes of ischemia and four hours of reperfusion. GI-Y1 pretreatment two hours before the onset of ischemia decreased the release of LDH and CK-MB and myocardial infarction as a percent of the risk zone by 30–35% compared to the untreated hearts. When GI-Y1 administration was delayed until one minute after the onset of ischemia, the infarct size was halved compared to the control hearts. GSDMD activation was inhibited by GI-Y1 in the infarcted myocardium, but not in the non-infarcted tissue. The results were quite similar when GSDMD knockout mice were evaluated [150,151]. After coronary occlusions of thirty to forty-five minutes and reperfusion for 24 h, less LDH was released and the infarction was 30–60% smaller in GSDMD^-/-^ mice than in wild-type mice.

Isolated mouse cardiomyocytes subjected to hypoxia/reoxygenation generate increased amounts of GSDMD compared to the control cells [152]. Furthermore, in open-chest mice undergoing thirty minutes of myocardial ischemia and three to twenty-four hours of reperfusion, the myocardial levels of both GSDMD and its N-terminal fragment were upregulated. The immunofluorescence analysis revealed the increased GSDMD accumulation occurred mainly in cardiomyocytes. Additionally, the infarct size in these hearts averaged 37% of the risk zone. When mouse hearts with the targeted deletion of the GSDMD gene were studied with identical protocols, the GSDMD protein was depleted in the myocardium, less LDH was released, and the infarct size of 20% was significantly smaller.

## 14. Caspase-1 Inhibition Is Additive to That of IPC

As described at length above, it is hoped that interventions using different modes of protection can be combined to achieve additional protection beyond that seen with any single treatment. This is also true for cas-1 antagonists. Untreated, open-chest rat hearts subjected to sixty minutes of coronary occlusion and then two hours of reperfusion demonstrated 60% infarction of the risk zone [143]. Ticagrelor administered ten minutes before reperfusion reduced infarction to 43%. And the cangrelor treatment itself resulted in 44% infarction. VX-765 injected by itself five minutes before reperfusion decreased the infarct size to 29% of the risk region. IPoC (three cycles of thirty-second occlusions and thirty-second reperfusions) diminished the infarction to 40%. The combination of cangrelor and VX-765 or ticagrelor and VX-765 further decreased infarction to 12 and 17%, respectively. Conversely, the combination of ticagrelor and IPoC was no more protective than either ticagrelor or IPoC alone. In our previous rat heart study, the combination of VX-765 and cangrelor had also shown a significant added protection when compared to either single intervention [144]. Finally, a subsequent intervention in isolated murine hearts revealed additive protection in those hearts treated with IPC and emricasan [146]. So, protection from P2Y_12_ antagonism or other conditioning maneuvers and cas-1 blockade is additive, as predicted.

To further document the presence of this additive protective effect of two interventions with distinctive modes of protection, we selected a murine cas-1 knockout model [146]. The mouse strain we obtained from Jackson Laboratory carried a knockout allele of the cas-1 gene, but also an incidental cas-4 deficiency unknowingly present in the embryonic stem cells used to develop the knockout model. Cas-4 is also capable of initiating pyroptosis. Whereas infarction in isolated wild-type mouse hearts averaged nearly 85% of the risk zone after fifty minutes of global ischemia and two hours of reperfusion, the infarcts were significantly smaller (approximately 42%) in the cas-1/4 knockout hearts. Not surprisingly, VRT-043198 had no additive protective effect in hearts lacking cas-1, whereas its protective effect in wild-type hearts was equivalent to the degree of protection observed in untreated knockout mice. Of particular interest was the apparent added protection when cas-1/4 knockout mice were exposed to three cycles of IPC. In the latter mice, infarction averaged 20% of the risk region. Therefore, in genetically intact rats and mice treated with pharmacologic cas-1 antagonists and in cas-1 genetically deficient mice and in both open-chest and isolated models, the addition of IPC or postconditioning agents, such as P2Y_12_ antagonists, unequivocally leads to additive protection against reperfusion injury.

As already described, calpeptin salvaged approximately one-third of the myocardium at risk after ischemia/reperfusion in isolated murine hearts [146]. As expected, emricasan, the pan-cas inhibitor, which was equally as protective as calpeptin, added no further protection when combined with calpeptin. Both agents decrease cas-1 activity; so, either’s salutary effect on reperfusion injury could not be supplemented by the other agent. It is noteworthy that emricasan’s protective effect was indeed magnified by the addition of IPC, resulting in the additional halving of infarction. Because of the critical importance of this concept of additive protection, we further explored the latter in the cas-1/4^-/-^ mice [146]. Mice lacking cas-1/4 have infarcts of approximately one-half of the size of those in wild-type mice. As expected, calpeptin, which salvages the ischemic myocardium in wild-type hearts, has no salutary effect when used to treat cas-1/4^-/-^ knockout mice. Again, both interventions target the same destructive element, cas-1. On the other hand, as previously noted, when these knockout mice hearts were exposed to IPC, infarction was again halved. Therefore, a wide range of pharmacologic agents and non-pharmacologic interventions can be combined to yield additive protection. The key is that the interventions being combined have different mechanisms of salvage.

Additive protection was also considered by Do Carmo et al. [145]. Rats were subjected to thirty-five minutes of coronary occlusion followed by two hours of reperfusion. An infusion of VX-765 initiated before ischemia and continued until the end of reperfusion decreased infarction from 48% of the risk zone in the control animals to 28%. Curiously, wortmannin, a PI3 kinase inhibitor, blocked this protective effect. More importantly, IPC, which by itself was quite protective, added little to the protection of VX-765. Although the average infarct size in the group treated with VX-765 and IPC fell to approximately 23%, this further diminution in the infarct size was not a significant change. It is possible that this contrary result may have been affected by the design of the experimental protocol. Do Carmo’s rats experienced only thirty-five minutes of coronary occlusion, whereas we exposed our experimental animals to fifty to sixty minutes of ischemia. With the shorter period of ischemia, a single protective intervention often decreased infarction to near or less than 20% so that the potential added protection of a second intervention might be difficult to detect. Longer periods of ischemia increase the infarction volume, making it easier to measure the added protective effects of second and even third interventions.

Penna et al. [80] also examined the possible additive protective effects of ticagrelor and NLRP3 inhibition in an isolated rat heart preparation. Rats were treated for three days with ticagrelor by oral gavage. Then, the hearts were removed and perfused on a Langendorff apparatus. The control hearts and hearts previously exposed to ticagrelor were perfused with buffer containing the NLRP3 inhibitor INF for twenty minutes before thirty minutes of global ischemia and sixty minutes of reperfusion. Ticagrelor decreased infarction from 70% of the risk region in the untreated rats to 50%. Infarction in the hearts treated only with INF averaged 38%. When the ticagrelor-treated hearts were exposed to INF, the infarct size averaged 44% of the risk region. Obviously, there was no additive protection, contrary to what we would have expected. Curiously, Penna et al. also noted that both the ticagrelor pretreatment and INF exposure prevented NLRP3 inflammasome activation and downstream signaling. Conversely, both ticagrelor pretreatment and INF exposure activated the RISK pathway. Hence, in these experimental animals, both the ticagrelor pretreatment and INF exposure stimulated the RISK pathway as well as blocked NLRP3 inflammasome activation. It is not known why each of the two agents had potent pharmacologic effects mimicking the main activity of the other intervention. However, it is clear that, if two interventions have similar effects on downstream signaling, there will not be any additive protection.

## 15. Concluding Remarks

Myocardial infarction results in the loss of functioning ventricular muscle. If enough muscle is lost, the onset of heart failure with its debilitating symptoms and shortened life expectancy ensue. Over the decades, many clinicians and scientists have proposed interventions that have failed to improve patient outlook. But this despair led to the advent of IPC, which finally proved that the heart could be made more resistant to infarction. The examination of IPC and later IPoC stimulated investigators to uncover the conditioning’s cell signaling and the events that inappropriately cause the death of cardiomyocytes after ischemia/reperfusion, thus permitting one to suggest ways to prevent it. The P2Y_12_ antagonists initially used to combat thrombosis are serendipitously also potent postconditioning agents. AMI patients are now routinely treated with these agents and may be benefiting from that protection. But, to offer that protection, the agents must be in the patient’s blood at the time of reperfusion. It is important to realize that protection may be blocked by ASA. This potential detrimental effect must be balanced against ASA’s ability to block thrombosis and promote early recanalization. The search for additional protective agents with different mechanisms of action has uncovered pharmacologic compounds that interfere with NLRP3 inflammasome assembly or directly block cas-1 or GSDMD activation. Finally, we do not know how many other targetable mechanisms contribute to the programed death of reperfused cardiomyocytes. Something is killing these cells. Experiments should test if protection from the blockade of any newly discovered process can be added to that of cas-1 inhibition, P2Y_12_ antagonism, and IPoC. If we could eliminate, or at least minimize, the death of cardiomyocytes from ischemia/reperfusion, debilitating AMI could become obsolete.
